# *Trifolium pratense* ethanolic extract alters the gut microbiota composition and regulates serum lipid profile in the ovariectomized rats

**DOI:** 10.1186/s12906-021-03486-w

**Published:** 2022-01-04

**Authors:** Yixian Quah, Na-Hye Park, Eon-Bee Lee, Ki-Ja Lee, Jireh Chan Yi-Le, Md. Sekendar Ali, Seung-Hee Jang, Min-Jeong Kim, Seung-Jin Lee, Seung-Chun Park

**Affiliations:** 1 College of Veterinary Medicine, Kyungpook National University, 80 Daehak-ro, Daegu, 41566 Republic of Korea; 2 Laboratory Animal Center, Daegu-Gyeongbuk Medical Innovation Foundation, Daegu, Republic of Korea; 3 Department of Finance, Faculty of Business and Finance, Universiti Tunku Abdul Rahman, Jalan Universiti, Bandar Barat, 31900 Kampar, Perak Malaysia; 4 Centre of IoT and Big Data, Universiti Tunku Abdul Rahman, 31900 Kampar, Perak Malaysia; 5 Department of Biomedical Science and Department of Pharmacology, School of Medicine, Brain Science and Engineering Institute, Kyungpook National University, Daegu, Republic of Korea; 6 Department of Pharmacy, International Islamic University Chittagong, Kumira, Chittagong, 4318 Bangladesh; 7 Teazen Co. Ltd., Gyegok-myeon, Haenam-gun, Jeollanam-do 59017 Republic of Korea; 8 Reproductive and Development Toxicology Research Group, Korea Institute of Toxicology, Daejeon, Republic of Korea

**Keywords:** Dysbiosis, Gut microbiota, Hypocholesterolemic effect, Menopause, *Trifolium pratense*

## Abstract

**Background:**

*Trifolium pratense* (red clover) ethanolic extract (TPEE) has been used as a popular over-the-counter remedy for the management of menopausal symptoms. Prolonged consumption of herbal extract has been shown to regulate the composition of gut microbiota. This study was designed to elucidate the influence of TPEE on the gut microbiota composition in the ovariectomized (OVX) rats.

**Methods:**

OVX rats were treated with TPEE at 125, 250, 500 mg/kg/day, or controls (pomegranate extract, 500 mg/kg/day; estradiol, 25 μg/kg/day) for 12 weeks. Gut microbiota analysis was conducted by extracting the microbial DNA from fecal samples and microbiome taxonomic profiling was carried out by using next-generation sequencing. The levels of serum biomarkers were analyzed using enzyme-linked immunosorbent assay (ELISA) kit. The prediction of functional biomarker of microbiota was performed using PICRUSt to investigate the potential pathways associated with gut health and serum lipid profile regulation. To study the correlation between gut microbiota composition and serum lipid levels, Spearman’s correlation coefficients were defined and analyzed. Additionally, gas chromatography–mass spectrometry analysis was conducted to uncover additional physiologically active ingredients.

**Results:**

TPEE-treated OVX rats showed significant reduction in serum triglycerides (TG), total cholesterols (TCHOL), and LDL/VLDL levels but increase in HDL level. The alteration in the pathways involve in metabolism was the most common among the other KEGG categories. Particularly, TPEE also significantly reduced the relative abundance of sequences read associated with inflammatory bowel disease (IBD) and the peroxisome proliferator-activated receptor (PPAR) signalling pathway. TPEE intervention was seen to reduce the Firmicutes to Bacteroidetes (F/B) ratio in the OVX rats, denoting a reduction in microbial dysbiosis in the OVX rats. Correlation analysis at the phylum level revealed that Bacteriodetes and Proteobacteria were strongly correlated with serum TG, TCHOL and HDL levels. At the species level, *Bifidobacterium pseudolongum* group was seen to positively correlate with serum HDL level and negatively correlated with serum AST, ALT, LDL/VLDL, TCHOL, and TG levels.

**Conclusions:**

TPEE treatment showed therapeutic benefits by improving the intestinal microbiota composition which strongly correlated with the serum lipid and cholesterol levels in the OVX rats.

**Supplementary Information:**

The online version contains supplementary material available at 10.1186/s12906-021-03486-w.

## Background

As the average life span of women increases globally, women would be spending longer extent of their lives in the postmenopausal stage. Therefore, research into major physiological changes associated with menopause is receiving increased clinical and commercial interest [1]. Menopause is diagnosed when menstrual cycles permanently cease due to the exhaustion of ovarian oocytes resulting from aging. The major consequences of menopause such as urogenital atrophy, osteoporosis, cancer, obesity, and cardiovascular diseases are related primarily to estrogen deficiency. Previous clinical findings indicate that the treatment of postmenopausal osteoporosis should also consider preventative measures for cardiovascular disease because osteoporotic postmenopausal women are at higher risk of suffering cardiovascular events [2]. This is because estrogens exert a protective role in the cardiovascular system and are produced primarily in the ovaries via a process that utilize low-density lipoprotein cholesterol (LDL) as a substrate. When a woman is experiencing menopause, there is a depletion of follicles production which leads to the reduction of estrogen level. The negative feedback mechanism of estrogen induces the secretion of follicle stimulating hormones (FSH) and causes the desensitization of the follicles towards FSH [3]. The follicle is left without the ability to utilize the circulatory LDL for the synthesis of estrogen thus give rise to decreased estrogen production and increased in blood LDL levels [4]. Conventional hormone replacement therapy have raised serious concerns about its side effects and safety when it was used in prolonged treatments in relation to cardiovascular events and hormone-related cancers [5]. Consequently, researchers are attempting to identify alternative therapeutics including nutrients and botanicals which are rich in phytoestrogen to improve the abovementioned postmenopausal symptoms.

*Trifolium pratense* L. (Fabaceae) (red clover) extract has been consumed in many countries including India [6], Italy [7] and Turkey [8] as traditional medicines. The native Americans used this plant to treat topical skin problems and complications in lungs, nervous and reproductive system [6, 9, 10]. In Pakistan, *T. pratense* was used to treat fever, sore throat, and meningitis [11]. In Kosovo, the juice of its leaves was used as remedy for stomach disorders [12]. While in Estonia, *T. pratense* was taken as recreational medicine to treat heart diseases [13]. In recent years, *T. pratense* has become a popular over-the-counter remedy for the management of menopausal symptoms. This dietary supplement has shown potential in the treatment of several conditions associated with menopause [14, 15]. Previous studies have also reported the effects of red clover extract and its active ingredients on vasomotor symptoms [14]. In addition, the benign effects of red clover extract have been reported in the breasts, endometrium, and neural structures [16]. Among the above multifunctional physiological activities, a meta-analysis on *T. pratense* reported that the plant have shown beneficial effects on the lipid profile of peri- and post-menopausal women [5].

A recent study demonstrated that women who have stopped menstruating have shown transformation in their compositional and functional features in their gut microbiota [17]. Although the mechanism of which is still unclear, estrogen has direct impact on the immune cells and variants in genes related to innate immunity and energy metabolism and thus this could cause the a shift in the gut microbiome [17]. Besides, the intestinal microbiome has been reported to play important role in isoflavone uptake, metabolism, and ultimately therapeutic efficacy [18]. Gut microflora metabolizes phytoestrogen daidzein, found in *T. pratense* extract [19], into equol which has high affinity for estrogen receptor (ER) β [20]. Thus, the interaction between plant derived phytoestrogens with gut microbiota could yield positive estrogenic effects mainly in tissues expressing ER β which mediates cholesterol metabolism [21, 22]. Prolonged consumption of herbal extract has been shown to regulate the composition of gut microbiota [23]. With an increasing understanding of human gut microbiota, it has become apparent that the microbiology of the gut is closely related to health and disease [23].

In short, the literatures pertaining to the effect of *T. pratense* in regulating the lipid profile and the effect of menopause in alteration of gut microbiota composition which regulates serum lipid profile strongly suggest that *T. pratense* could regulate intestinal microbiota and serum lipid profile in menopausal women. TPEE-treated OVX rats could provide insights about the interrelationship between serum lipid profile and the changes of intestinal microflora.

## Methods

### Preparation of ethanolic extract from *Trifolium pratense* and species identification

Dried *T. pratense* was taxonomically identified by Dowgene DNA Testing Company (Seoul, Republic of Korea) using mitochondrial DNA sequencing. The DNA sequencing data from our study was 100% identical to previously reported sequences of *T. pratense* in the NCBI GenBank database with the accession numbers: KY860927.1, KX555647.1, KU956946.1, FJ554421.1, DQ312029.1, and DQ307475.1 (Additional File 1). The raw sequence data was deposited to Sequence Read Archive (SRA) with accession number: SRR14492033, project number: PRJNA727441.

We obtained the species identified *T. pratense* from a certified company, Teazen Co. Ltd. (Anyang-si, Republic of Korea), which was cultured and originated from Albania (Certificate of Origin Number A19690728 was issued on 2019-02-11 by Industrie- und Handelskammer Würzburg-Schweinfurt, verification code: GSY4-3M2C-CP9C), with the voucher number P-338852. The relevant institutional permissions to collect *T. pratense* was obtained. This study complies with the with local and national regulations. The extraction was done by DaeHo Co. Ltd. (Gyeonggi-do, Republic of Korea), abiding to GMP standards and the Food Item Manufacturing Report. Briefly, macerated *T. pratense* leaves and flowers (30 kg) were added to extraction solvent (30% ethanol) at 1:30 ratio. The sample was extracted twice for 3 h at 85 °C; the extract obtained from each extraction step was filtered by 1-μm filtration. The resulting *T. pratense* ethanolic extract (TPEE) was then incorporated with dextrin at a 7:3 ratio (extract:dextrin) during a spray-drying step at 180 °C for animal experiment as the prototype of a product (Additional file 2). The extract was standardized and managed by two functional indicator components: formononetin and biochanin A (Additional file 2). HPLC quantification of the indicative compounds (formononetin and biochanin A) in TPEE was conducted and reported in our previous study [24].

### GC-MS analysis

In addition to the known functional ingredients of *T. pratense*, GC-MS analysis on the components in this plant was conducted to uncover additional physiologically active ingredients. TPEE was dissolved in 70% ethanol at 1 mg/mL. GC analysis was performed on Agilent 7890A GC and 5975C MSD equipped with injector (250 °C) and a flame ionization detector (FID). Helium gas was used as carrier gas (1 mL/min) and the capillary column used was DM-5 ms (60 m × 250 μm, film thickness 0.25 μm). The column temperature was kept at 60 °C for 1 min and then heated to 300 °C with a 5 °C/min rate and kept constant at 300 °C for 20 min. GC-MS analysis was performed using a GC-MSD. The column temperature was kept at 60 °C for 1 min and programmed to 300 °C at a rate of 5 °C/min and kept constant at 300 °C for 20 min. The flow rate of helium as carrier gas was 1 mL/min. The mass range for GC-MS was 35–600 (m/z). MS were taken at 70 eV. HPLC quantification of the indicative compounds (formononetin and biochanin A) in TPEE was conducted and reported in our previous study [24].

### Animal grouping and treatments

Healthy 8-week-old female Sprague–Dawley rats (body weight: 190–230 g) were housed in an air-conditioned environment (22 °C ± 2 °C, humidity of 50–60% with a 12 h light/dark cycle with lights on at 6:00 A.M). The animals were allowed free access to Teklad certified irradiated global 18% protein rodent diet (2918C, Envigo, USA) supplied by Koatech (Gyeonggi-do, Republic of Korea) and distilled water. Animal handling complied with ARRIVE guidelines and was performed in compliance with the Guide for the Care and Use of Laboratory Animals provided by the US National Institutes of Health. This study was approved by the institutional animal care and use committee of the Kyungpook National University, Republic of Korea (approval number: KNU 2018–121). A total of 70 rats were acclimatized for 1 week. In this study, n refers to number of animals. The rats were randomly allocated as either Sham-operated (*n* = 10) or bilaterally ovariectomized (OVX; *n* = 60). Oral administration of sample, positive controls or vehicle to the rats began 1 week after surgery. The experimental timeline was shown in Additional file 3.

The OVX rats were randomly divided into six groups and each group was composed of 10 rats. The group were named after the treatment they received: Negative control (NC) = OVX + 30% dextrin; Pomegranate extract (PomE) = OVX + 500 mg/kg/day pomegranate extract; Estradiol (E) = OVX + 25 μg/kg/day estradiol; T125 = OVX + 125 mg/kg/day TPEE; T250 = OVX + 250 mg/kg/day TPEE; T500 = OVX + 500 mg/kg/day TPEE.

Sham and NC groups were administered the same volume of 30% dextrin by oral gavage. Three controls were as follows: Sham group (representing the presence of endogenous estradiol in the body), E group (administration of estradiol, a positive control drug used for hormone replacement therapy in the treatment of postmenopausal osteoporosis [25]) and PomE group (pomegranate extract as a plant extract positive control for osteoporosis [26, 27] and cardiovascular disease (ClinicalTrials.gov Identifier: NCT01102140) intervention). PomE was purchased from Hanil PFC Co., Ltd. (Seoul, Republic of Korea), which was approved and recognized by MFDS (Ministry of Food and Drug Safety) as health functional food. Chemical characterization of the PomE has been carried out with LC/MS/MS and was reported in previous report [28].

At the end of the treatment, the rats were anesthetized with CO_2_ inhalation until unconscious and blood samples were taken via cardiac exsanguination. The blood samples were centrifuged for 10 min at 1000 x *g* to obtain serum and then stored at − 80 °C until use. The fecal samples were collected and stored at − 80 °C until DNA extraction for microbiota profiling.

### Preparation of specimens

The harvested liver samples were immediately fixed in 10% buffered formalin solution. Paraffin embedded blocks (5 μm) were stained with hematoxylin and eosin (H&E) for histology analysis under an optical microscope.

### Serum biomarker analysis

Serum for biomarker analysis were maintained at − 80 °C until the respective tests were conducted. Levels of serum biomarkers were measured using an enzyme-linked immunosorbent assay (ELISA) kit (Cusabio Biotech, Wuhan, China). Sample analysis and calibration curves were plotted according to the manufacturer’s instructions.

### Min–max normalization

To allow direct comparison among the data obtained from different analysis in our study, a metric system was constructed. The data obtained in each analysis were normalized using Min-Max normalization [24] to ensure the output was bounded between 1 and 5. Specifically, the NC was scored as 1 and the Sham as 5. The general formula of the Min-Max normalization is as follows:

$x^{\prime }=\left(\frac{x-\min (X)}{\max (X)-\min (X)}\right)$, where x ∈ X, X are real numbers.

### Gut microbiota analysis

Group NC, Sham, E and T250 were selected for the elucidation for their gut microbiota composition. Group T250 was selected to be the representative for the TPEE treatment groups due to the lack of significance differences among the three treatment dosages in the serum biomarker levels. Microbial DNA from fecal samples was extracted using a QIAamp DNA Stool Mini Kit (Qiagen Inc., Hilden, Germany) according to the manufacturer’s instructions. Quality control requirement of the DNA sample was met by achieving DNA concentration of ≥15 ng/μL and A_260_/A_280_ ratio of ≥1.8. Microbiome profiling was conducted based on 16S rRNA gene sequences obtained from the rats’ fecal sample, it was amplified by universal bacterial PCR primers 27F (AGRGTTTGATYMTGGCTCAG) and 1492R (GGYTACCTTGTTACGACTT) by the Pacific Biosciences (PacBio) RS1 conducted in ChunLab Inc. (Seoul, Republic of Korea). Microbiome Taxonomic Profile (MTP) was generated from next generation sequencing (NGS) using the EzBioCloud MTP pipeline and EzBioCloud 16S database PKSSU4.0 [29]. Sequences were separated by unique in-house codes, and low- quality reads (sequences with the read lengths of < 100 or > 2000 bp; average Q values < 25, and sequences that do not match with any of the reference sequences with ≤97% similarity cutoff were clustered using UCLUST with 97% similarity cutoff) were filtered out. The number of reads (total reads pre-filter, total valid reads, filtered reads) and the read lengths for each sample were presented in Additional file 4. The species identified, query sequences achieved 97% similarity with the EzBioCloud 16S database and the OTUs obtained by those which did not achieve 97% similarity with the database (which then clustered using UCLUST tool) were used to generate a final set of OTUs for alpha diversity indices (including Shannon and Chao1 indices). Secondary analyses including beta diversity and functional biomarker discovery were analyzed using EzBioCloud 16S-based MTP app [29]. The prediction of functional biomarker of microbiota was performed using the PICRUSt (ver. 1.0.0) [30].

### Statistical analysis

Data are expressed as means ± standard error of the mean (SEM), and statistical significance (*p* < 0.05) was determined by one-way ANOVA with Tukey’s post-hoc analysis using GraphPad Prism 5.01 (La Jolla, CA, USA). The significantly different KEGG pathway was selected by corrected *p* value < 0.05 using Kruskal-Wallis H-test with Benjamin-Hochberg correction.

## Results

### GC-MS analysis of *Trifolium pratense* ethanolic extract

**Fig. 1:**
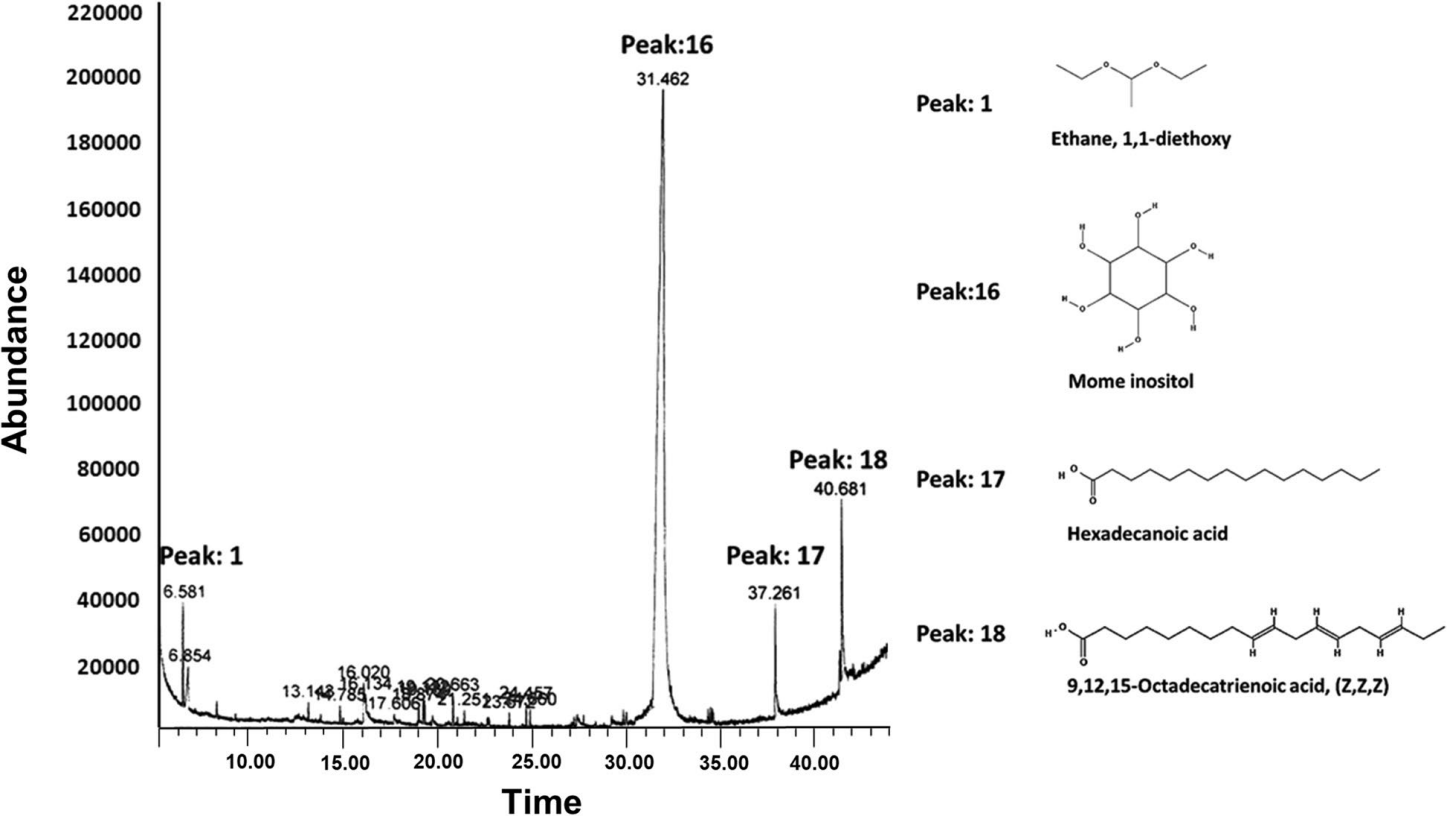
Chemical composition analysis of *Trifolium pratense* ethanolic extract (TPEE). The representative GC-MS chromatogram of TPEE was shown. The peaks of major compounds namely ethane, 1,1-diethoxy- (peak 1), mome inositol (peak 16), hexadecanoic acid (peak 17), and 9,12,15-octadecatrienoic acid, (Z,Z,Z)- (peak 18) found in TPEE were indicated

GC-MS analysis of TPEE led to the identification of 18 functional phytocomponents (Table 1). Particularly, the main constituents of TPEE identified by GC-MS analysis were mome inositol (68.21%), 9,12,15-octadecatrienoic acid, (Z,Z,Z)- (3.20%), hexadecanoic acid (1.92%), silane, diethoxydimethyl- (1.32%), ethane, 1,1-diethoxy- (1.14%), and 1-Butanol, 3-methyl-, acetate (1.03%) (Fig. 1). Remarkably, mome inositol (C_7_H_14_O_6_) was the most abundant in this extract.

**Table 1:** Phytocomponents identified in *Trifolium pratense* ethanolic extract by GC–MS

Peak	RT	Compound	Area %
1	6.581	Ethane, 1,1-diethoxy-	1.135
2	6.854	Silane, diethoxydimethyl-	1.321
3	13.143	Methoxyacetic acid, 2-tridecyl ester	0.281
4	14.785	Methoxyacetic acid, 2-tetradecyl ester	0.257
5	16.020	1-Butanol, 3-methyl-, acetate	1.026
6	16.134	Silane, trichlotodocosyl-	0.612
7	17.606	4-Amino-1,2-thiazole-3-carboxylic acid	0.252
8	18.874	Nonyl heptafluorobutyrate	0.183
9	19.112	Dodecane	0.308
10	19.189	Methyl salicylate	0.333
11	20.663	2-Methyl-4-(4-methoxiphenyl)-2-pentene	0.463
12	21.251	Methoxyacetic acid, 2-tetradecyl ester	0.233
13	23.572	Eugenol	0.215
14	24.457	9-Tricosene, (Z)-	0.221
15	24.660	Tetradecane,2,6,10-trimethyl-	0.227
16	31.462	Mome inositol	68.206
17	37.261	Hexadecanoic acid	1.924
18	40.681	9,12,15-Octadecatrienoic acid, (Z,Z,Z)-	3.198

### Effects of *Trifolium pratense* ethanolic extract on serum biomarker levels

**Fig. 2:**
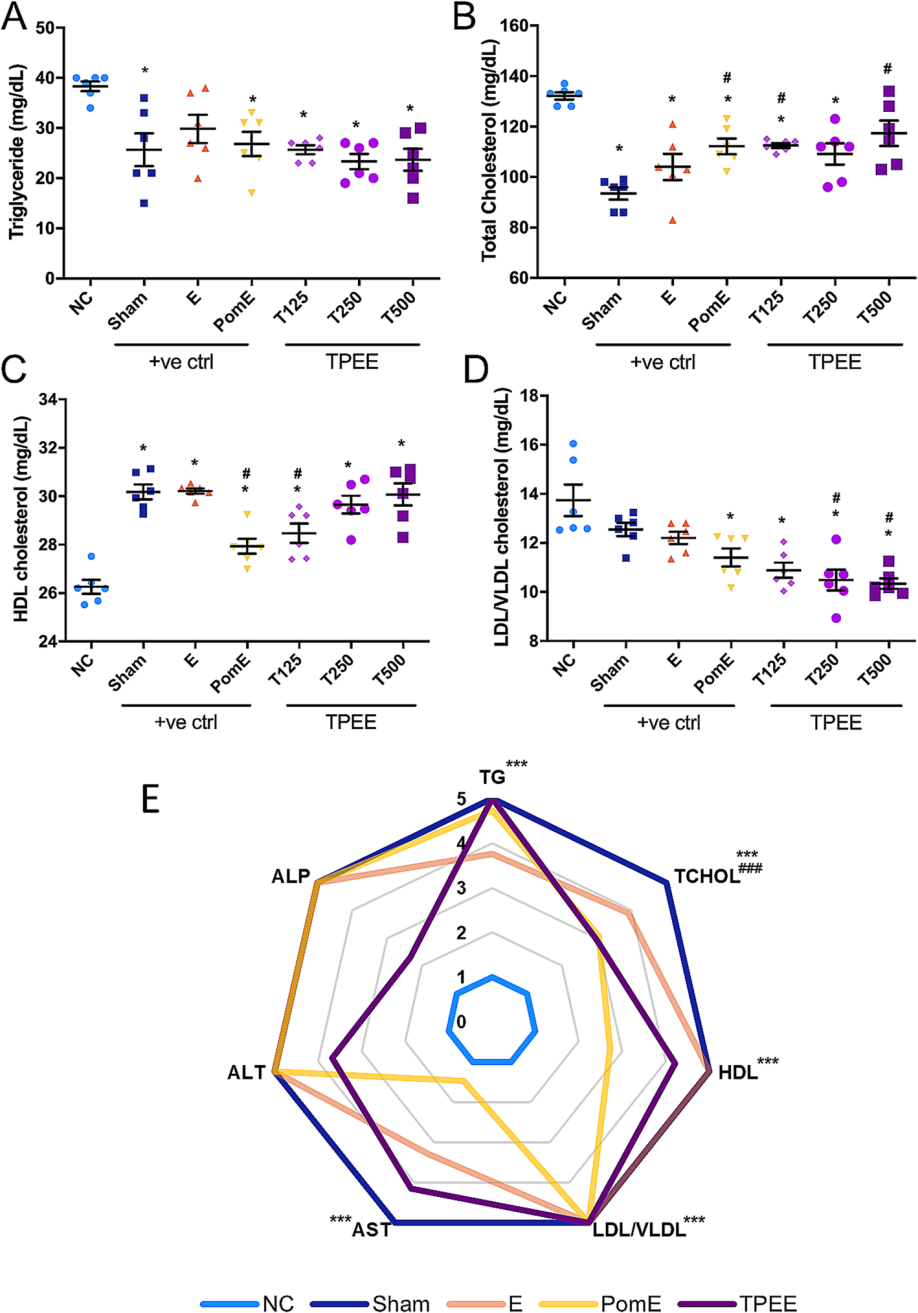
The effects of *Trifolium pratense* ethanolic extracts on the serum lipid profiles. Serum triglyceride (**A**), total cholesterol (**B**), HDL cholesterol (**C**) and LDL/VLDL cholesterol (**D**) levels in ovariectomized (OVX) rats were shown. Data are presented as means ± SEM, *n* = 6. ^*^p < 0.05 compared with the negative control (NC) group, ^#^*p* < 0.05 compared with the positive control (Sham) group determined by one-way ANOVA with Tukey’s post-hoc analysis. Summary of serum lipid markers and liver enzymes with the negative control (NC) scored as 1 and Sham scored as 5 (**E**). Data are presented as means ± SEM, *n* = 6. ^***^p < 0.001 TPEE compared with the negative control (NC) group, ^###^*p* < 0.001 TPEE compared with the positive control (Sham) group determined by one-way ANOVA with Tukey’s post-hoc analysis

Serum biomarker analysis revealed that TPEE treatment significantly reduced serum triglyceride (TG) and low-density lipoprotein/very low-density lipoprotein **(**LDL/VLDL) levels in OVX rats while significantly increased serum high-density lipoprotein (HDL) levels in OVX rats in a dose-dependent manner (Fig. 2). Administration of TPEE to OVX rats did not significantly affect AST, ALT, ALP and TBIL levels compared to NC and Sham groups (*p* > 0.05). However, there were significant reductions (*p* < 0.05) in the levels of TP and ALB (Table 2). The histology analysis on liver did not show significant changes in the treatment groups compared to the control groups (Additional File 5). From the scoring of the serum lipid markers and liver function indicators shown in Fig. 2E, TPEE showed promising positive effects on regulating serum cholesterol levels compared to PomE treatment (a marketed plant-based positive control). However, its effects on the liver function indicators were slightly weaker compared to that of E and PomE treatments.

**Table 2:** Effect of *Trifolium pratense* ethanolic extract on the levels of serum biomarkers

Group	AST (U/L)	ALT (U/L)	ALP (U/L)	TP (g/dL)	ALB (g/dL)	TBIL (mg/dL)
**NC**	269.00 ± 65.00	72.00 ± 2.90	247.00 ± 11.00	6.47 ± 0.09	4.00 ± 0.04	0.07 ± 0.02
**Sham**	207.00 ± 16.00	72.00 ± 1.90	246.00 ± 18.00	6.15 ± 0.12^*^	3.90 ± 0.06	0.03 ± 0.02
**E**	234.00 ± 25.00	90.00 ± 9.00	223.00 ± 29.55	5.88 ± 0.05^*^	3.60 ± 0.03^*#^	0.02 ± 0.02
**PomE**	262.00 ± 18.00	99.00 ± 2.20^*#^	227.00 ± 15.15	5.65 ± 0.08^*#^	3.70 ± 0.05^*#^	0.02 ± 0.02
**T125**	228.00 ± 9.30	80.00 ± 4.80	235.30 ± 16.43	5.73 ± 0.02^*#^	3.60 ± 0.02^*#^	0.05 ± 0.02
**T250**	226.00 ± 22.00	87.00 ± 5.40	278.70 ± 6.766	5.67 ± 0.07^*#^	3.60 ± 0.03^*#^	0.05 ± 0.02
**T500**	143.00 ± 18.00^*^	66.00 ± 4.00	290.50 ± 17.16	5.88 ± 0.04^*^	3.60 ± 0.03^*#^	0.03 ± 0.02

Values are presented as mean ± Standard error, *n* = 6

*AST* aspartate transaminase, *ALT* alanine transaminase, *ALP* alkaline phosphatase, *TP* total protein, *ALB* albumin, *TBIL* total bilirubin

^*^p ≤ 0.05 compared with the NC group

^#^*p* ≤ 0.05 compared with the Sham group

### Influence of *Trifolium pratense* ethanolic extract on the composition of gut microbiota of OVX rats

#### Effects of *Trifolium pratense* ethanolic extract on the diversity of the gut microbiota

**Fig. 3:**
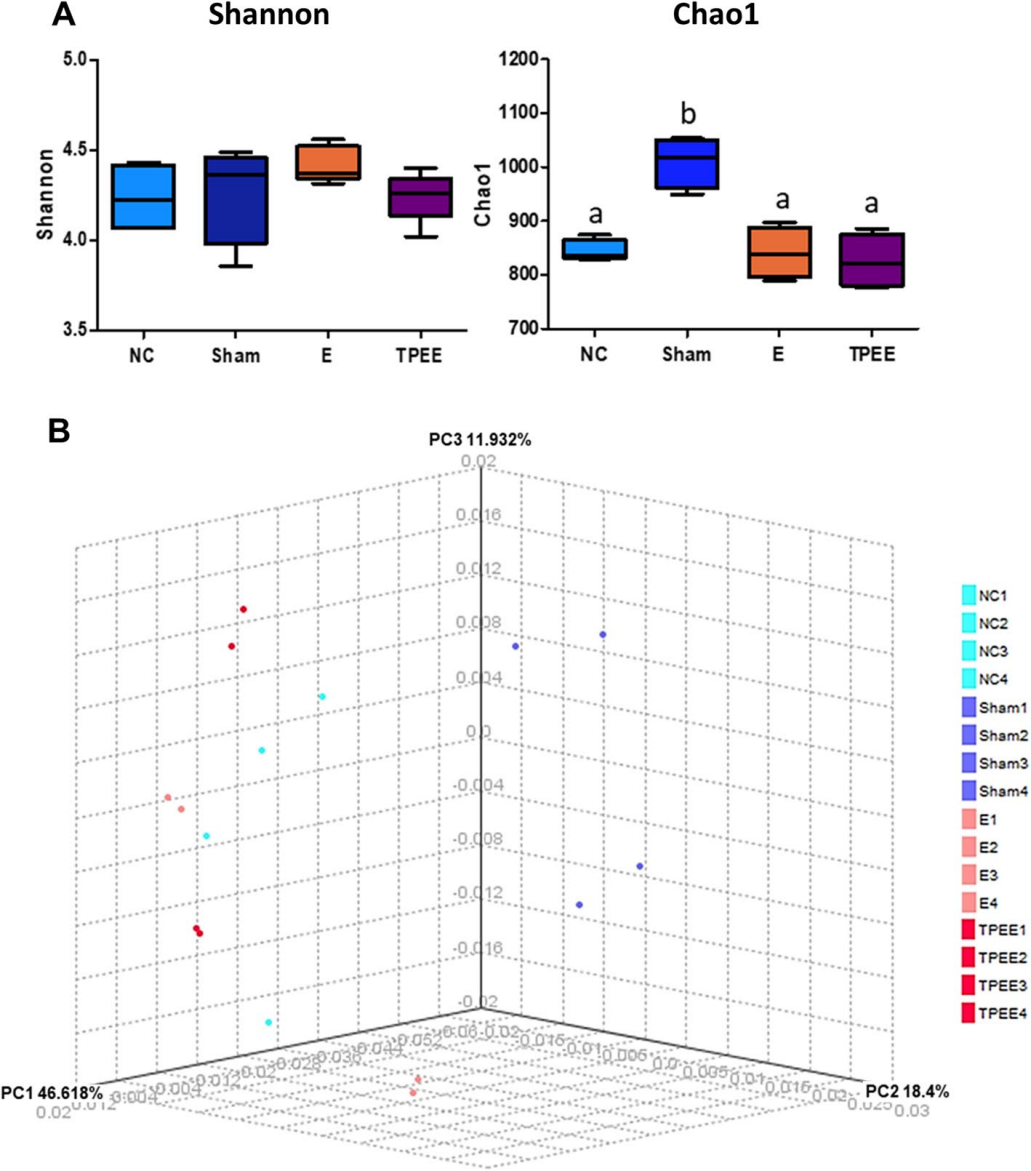
The alpha and beta diversity analyses of each treated group. Shannon and Chao1 index (**A**); beta diversity indicated by PCoA plot of weighted UniFrac distance showing sample clustering by treatment groups (**B**). Data are presented as means ± SEM, *n* = 4. Means denoted by a different letter indicate significant differences between treatments (*p* < 0.05) determined by one-way ANOVA with Tukey’s post-hoc analysis

The alpha diversity of the gut microbiota in the rats were assessed by Shannon and Chao1 diversity indices (Fig. 3A). The Shannon diversity index indicated that there was no significant difference among all the groups (*p* > 0.05). Interestingly, significant alteration in the Chao1 index was observed in the Sham group compared to the OVX groups (*p* < 0.001). Rarefaction curve was presented to illustrate the number of OTUs has reached a horizontal asymptote. This implies that the sequencing depth is sufficient for analysis (Additional file 6).

#### Beta diversity distance analysis

Weighted Fast UniFrac analysis was performed (Fig. 3B) and the data of the UniFrac matrices were projected on to a 3-dimensional plot using principal coordinates analysis (PCoA). Weighted PCoA analysis showed that most of the OVX samples (E, NC and TPEE) were largely indistinguishable and clustered closely to each other regardless of treatments. Sham group, on the other hand, has shown distinct clustering from the OVX samples. Visible OVX-to-Sham variation was observed which was largely distributed at principal coordinate 1 (PC1) of 46.618%.

**Fig. 4:**
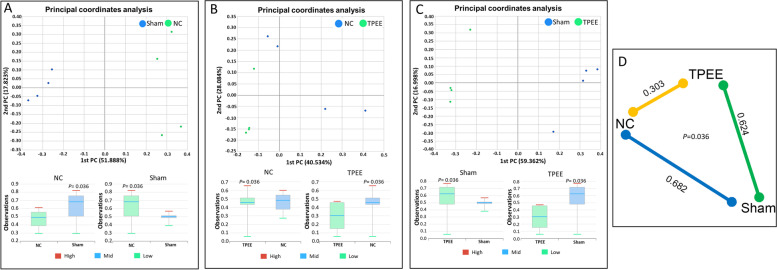
Bray-Curtis diversity distance analysis. The gut microbiota among the groups were compared using the PCoA plot (**A-C**). The summary of distances between NC, TPEE and Sham groups were shown (**D**)

Bray-Curtis distance analysis was performed to provide further information on similarities and differences of gut microbiota composition between and within groups. The diversity distance was performed to compare TPEE with the NC and Sham groups. Figure 4A-C illustrate that the median of inter-group distance between NC, Sham and TPEE were generally larger than intra-group distance. Figure 4D illustrates the summary of the median distance among the three groups (NC, TPEE and Sham groups). The Bray-Curtis diversity distance analysis showed that the gut microbiota diversity in OVX rats drifted under the TPEE treatment with the median distance of 0.303 from the NC group. The median distance between Sham and TPEE (0.624) was seen to be slightly closer than distance between Sham and NC (0.682). Permutational multivariate analysis of variance (PERMANOVA) analysis showed that the inter-group distances were significantly different at 5% confidence interval (*p* = 0.036).

#### Predicted functions of gut microbiome

**Fig. 5:**
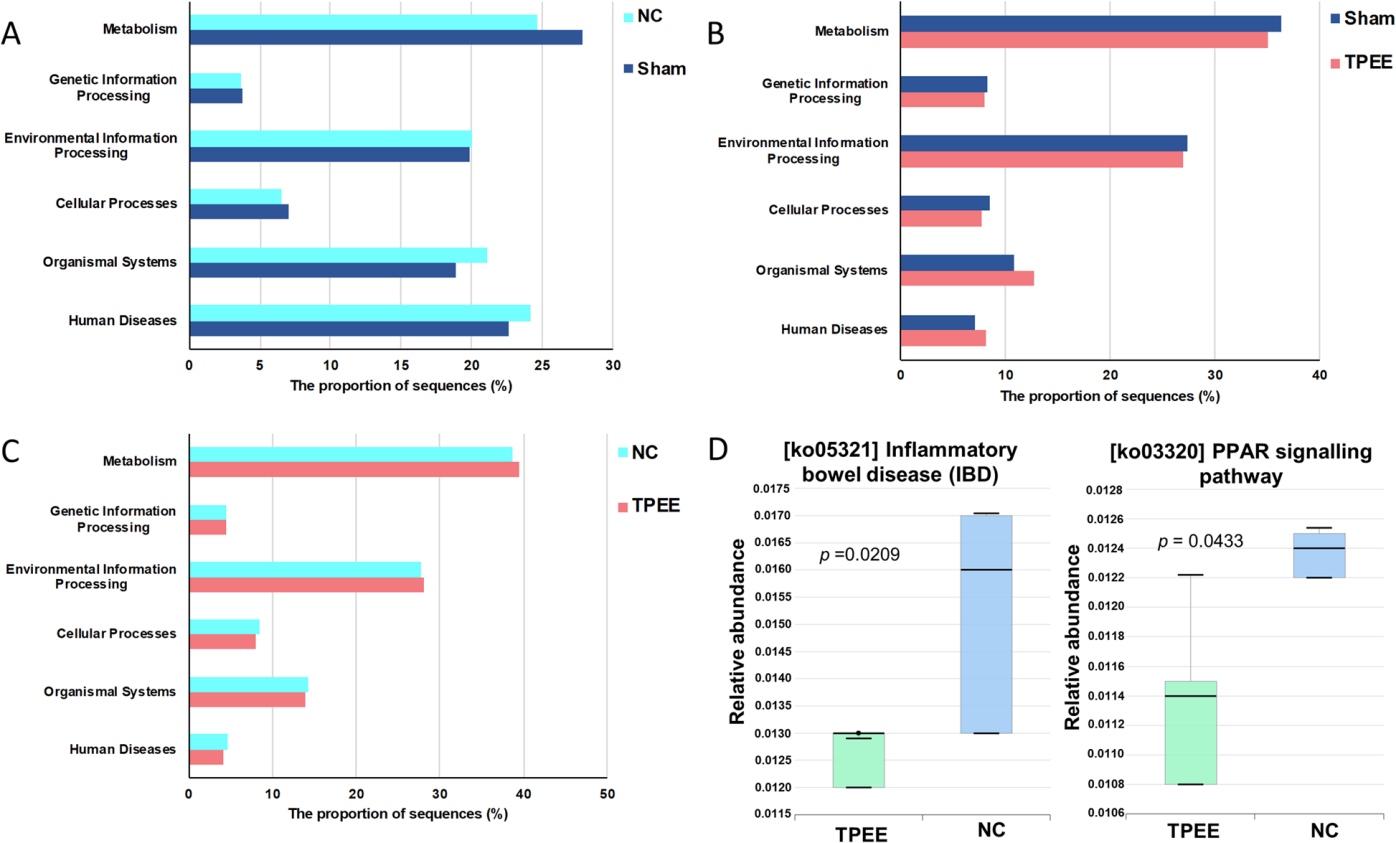
Comparison of predicted KEGG pathway between NC, Sham and TPEE groups using PICRUSt. The proportion of sequences of NC versus Sham (**A**), Sham versus TPEE (**B**), and NC versus TPEE (**C**). The box plot represents the distribution of the relative abundance of gut microbiome in TPEE and NC groups based on the predicted functional biomarkers associated with inflammatory bowel disease (IBD) and peroxisome proliferator-activated receptor (PPAR) signalling pathways (**D**). The box plot presented the median with the maximum and minimum values corrected by *p* < 0.05 determined by Kruskal-Wallis H-test with Benjamin-Hochberg correction

The function of gut microbiota was predicted by KEGG pathways using PICRUSt [31]. The significantly different pathway was selected by corrected *p* value < 0.05 using Kruskal-Wallis H-test with Benjamin-Hochberg correction. In Fig. 5, the proportion of sequence reads associated with metabolism pathway was the highest among the pathway classes. When comparing TPEE with Sham and NC groups, the proportion of sequences related to environmental information processing was the second highest, followed by organismal systems. Two metabolic pathways related to gut health and serum lipid profiles were selected for further investigation (Fig. 5D). The median relative abundance of the gut microbiome in the TPEE group associated with inflammatory bowel disease (IBD) was 0.013 while NC group was 0.016 (*p* = 0.0209). The significant orthologs that are in this pathway were transcription factor p65 (RELA), transcription factor Maf (c-maf), and toll-like receptor 4 (TLR4) with the KEGG orthology (KO) of K04735, K09035, and K10160, respectively. The median relative abundance of the gut microbiome in the TPEE group associated with peroxisome proliferator-activated receptor (PPAR) signalling pathway was 0.114 while NC group was 0.124 (*p* = 0.0433). The significant orthologs that are in the PPAR signalling pathway were acyl-coA oxidase (K00232), stearoyl-coA desaturase (SCD-1) (K00507), glycerol kinase (GyK) (K00864), integrin-linked kinase (ILK) (K06272), retinoid-X receptor beta (RXR) (K08525), and apolipoprotein A-I (APOA1) (K08757).

#### Correlations of serum biomarkers and gut microbiota

**Fig. 6:**
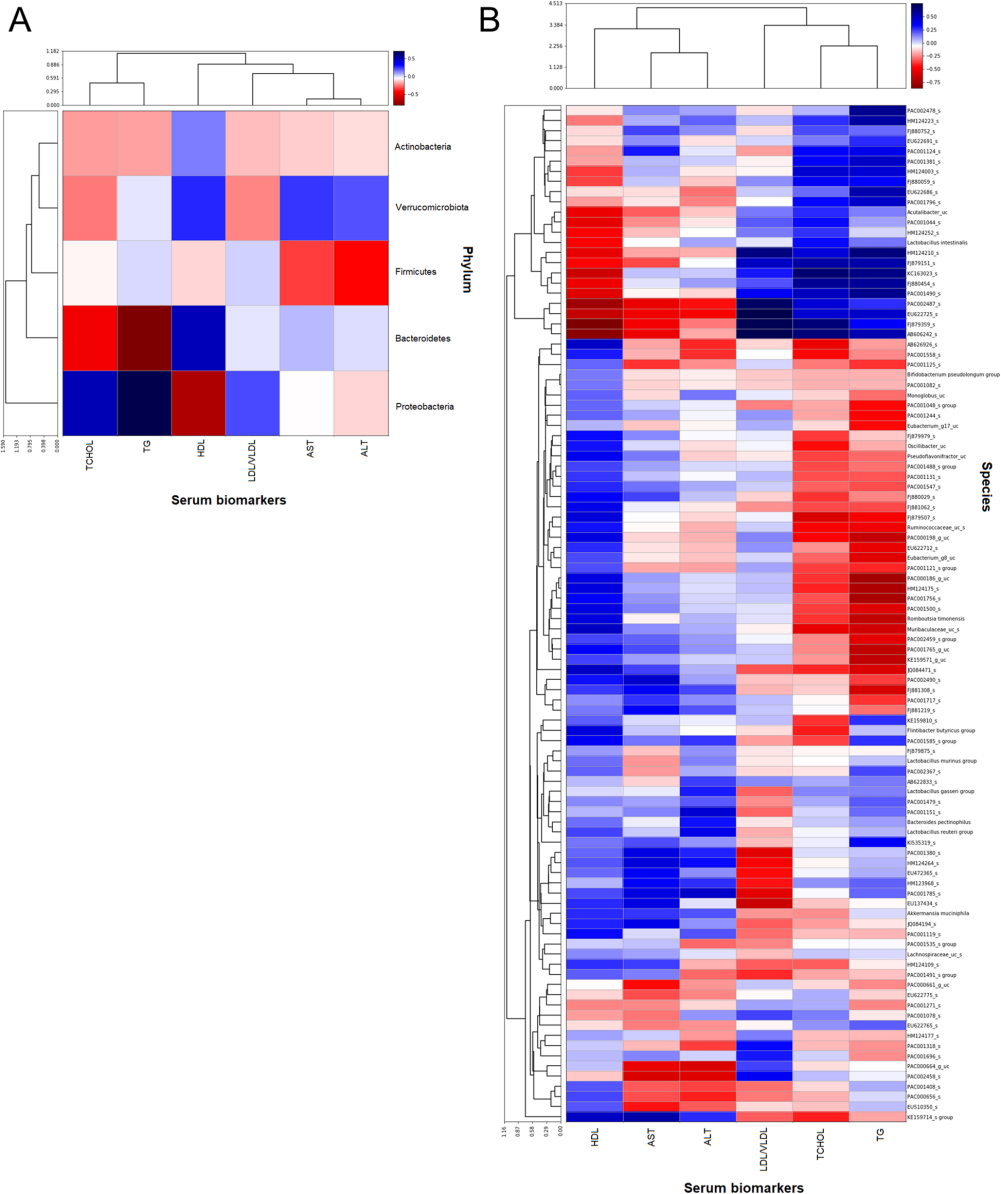
Heat map created and sorted based on hierarchical clustering of both bacteria and serum biomarkers. Dendrogram at the y-axis indicated the clustering of gut microbiota at the phylum level (**A**) or species level (**B**) in relation to each of the serum biomarkers. Dendrogram at the x-axis indicated the association clustering of serum biomarkers in relation to each phylum or species

Spearman’s correlation coefficients between the composition of gut microbiota and lipid biomarkers in OVX rats at the phylum (Fig. 6A) and species (Fig. 6B) levels were defined and hierarchical clustering was applied using Ward’s linkage. The heatmap illustrated that Bacteriodetes correlated negatively with serum TG and TCHOL level while correlated positively with serum HDL level (Fig. 6A). Conversely, Proteobacteria correlated positively with serum TG, TCHOL and LDL/VLDL level while negatively with serum HDL level. *Bifidobacterium pseudolongum* group was seen to positively correlated with serum HDL level and negatively correlated with serum AST, ALT, LDL/VLDL, TCHOL, and TG levels (Fig. 6B).

#### TPEE-altered intestinal microbiota composition in OVX rats

**Fig. 7:**
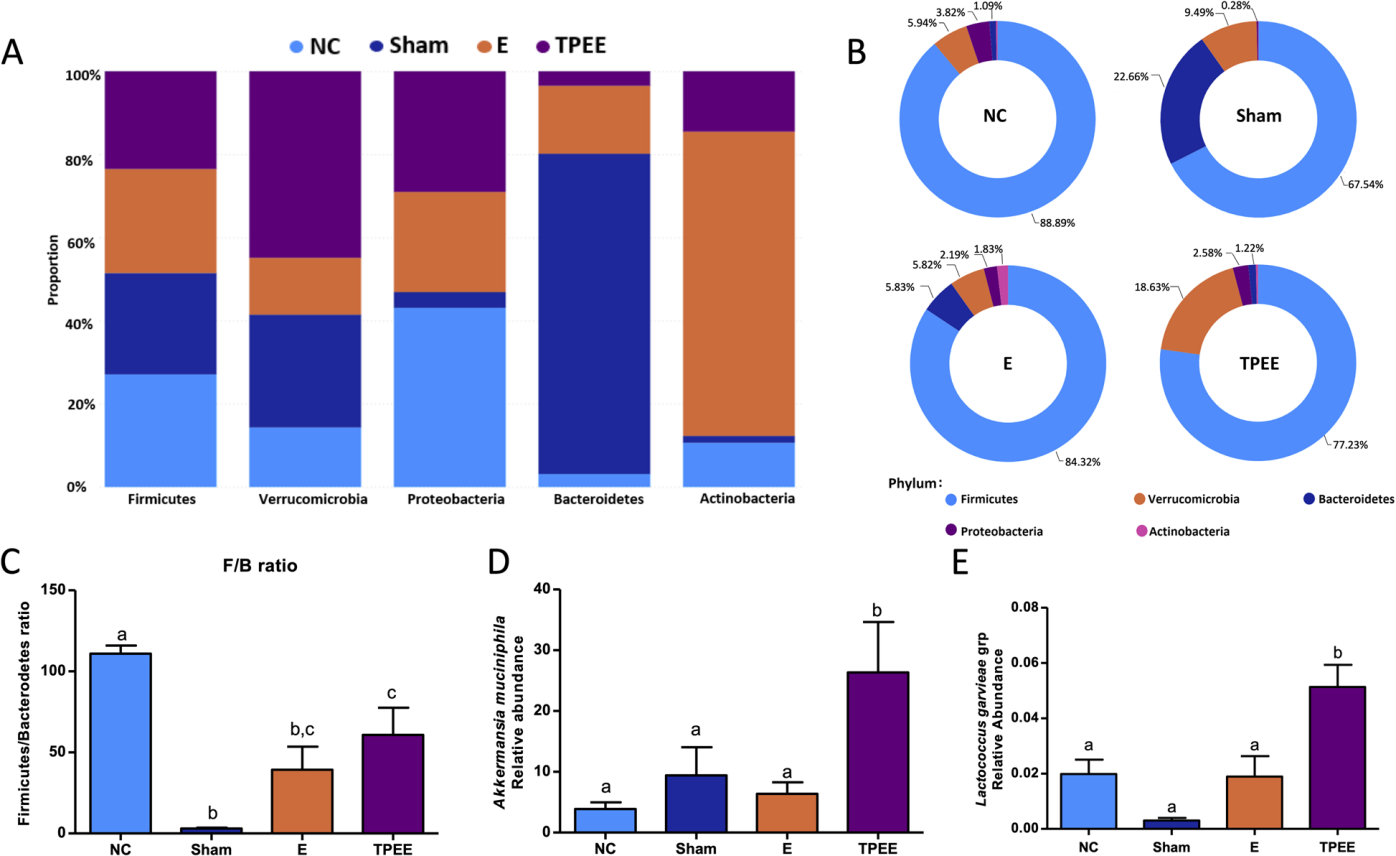
The composition of gut microbiota at the phylum level (**A-B**). The Firmicutes/Bacterodetes (F/B) ratio in each animal group (**C**). The relative abundance of *Akkermansia muciniphila* (**D**) and *Lactococcus garvieae* (**E**) in the different treatment groups were shown. Data are presented as means ± SEM, *n* = 4. Different letters indicate statistically significant differences at *p* < 0.05 determined by one-way ANOVA with Tukey’s post-hoc analysis

From our taxonomic analysis, the gut microbiota of rats consisted of Firmicutes, Proteobacteria, Verrucomicrobia, Actinobacteria, and Bacteroidetes at the phylum level. Firmicutes was the most abundant phylum (found in all the animals), followed by Verrucomicrobia and Bacteroidetes (Fig. 7A and B). TPEE intervention significantly improved intestinal microbiota composition in OVX rats by recovering an abundance of Verrucomicrobia (18.63%) (*p* < 0.05) and reducing Firmicutes abundance (77.23%) (*p* < 0.05) compared to the NC group (5.94 and 88.89%, respectively). Bacteroidetes were noticeably more abundant in the Sham group (22.6%) than in NC (1.09%, *p* < 0.05), E and TPEE (5.83, 1.22%, respectively) (*p* > 0.05). The Firmicutes/Bacteroidetes (F/B) ratio was found to be largely reduced in Sham group compared to NC group (*p* < 0.01) as shown in Fig. 7C. Treatment with E and TPEE in OVX rats significantly reduced the F/B ratio compared to that of NC group. No significant difference was observed between E, TPEE and Sham group in terms of F/B ratio. The strain with the highest relative abundance in the taxonomic composition analysis was *Akkermansia muciniphila*. Its abundance was highest in the TPEE group relative to the E and NC groups (Fig. 7D). In the present study, the relative abundance of *Lactococcus garvieae* was significantly higher in TPEE-treated groups than that in the NC group (Fig. 7E).

## Discussion

Red clover extract has been consumed as a dietary supplement for handling menopausal symptoms, cholesterol levels and osteoporosis [14, 15, 32]. Long term consumption of herbal extracts can shape the distribution and composition of the gut microbiota [23]. Therefore, it is important to study the interaction between gut microbiota and the administration of TPEE. In this study, TPEE and exogenous estrogen treatment did not significantly affect the gut microbiota species evenness and richness in OVX rats. This suggests that exogenous estrogen and TPEE treatments, known to contain phytoestrogens, could not reverse the decline in microbial diversity caused by overiectomization. However, TPEE administration have positively affected the population of beneficial bacteria strains that may contribute to the regulation of serum lipid levels.

Prediction of functional biomarkers showed that pathways associated to metabolism, genetic information processing, organismal systems and human diseases were found to alter with the influence of TPEE in the OVX rats following the same pattern as Sham (Fig. 5A-C). Particularly, TPEE treatment was found to yield lower abundance of sequence reads associated with IBD compared to the NC group (Fig. 5D). IBD is characterized by chronic inflammation of the intestine due to environmental and genetic factors, infectious microbes, and the dysregulated immune system [33], which includes Crohn’s disease and ulcerative colitis. The significant KOs found in this pathway, namely TLR4 and RELA, involve in the toll-like receptor signalling pathway which leads to inflammation [34]. Besides, RELA also plays a role in the cytokine-cytokine receptor interaction in Th1 lymphocyte to induce IFN-γ expression [35]. IFN-γ was known to induce intestinal vessels’ permeability by disrupting the vascular endothelial cadherin junction which associated with tissue injury and progression of Crohn’s disease [36]. Therefore, our result signifies that TPEE treatment could reduce inflammatory responses in the gastrointestinal tract and improve gut health in OVX rats through the possible reduction of TLR4, RELA and c-maf in the IBD associated pathways.

PPARs belong to the nuclear hormone receptors that are activated by fatty acids and their derivatives. PPARs play a critical role in modulating the gene expressions which involves in the glucose and lipid metabolism, adipogenesis and inflammation [37, 38]. PPAR signaling pathway was predicted to be lower in the TPEE treated group than in the NC group. The significant KOs discovered in this pathway were composed of SCD-1 and GyK genes, which associated with lipogenesis and gluconeogenesis, respectively. These KOs were modulated by one of the PPAR subtypes, PPAR γ, which involves in lipid metabolism. Another significant KOs determined in this pathway were ILK and APOA1 genes, they are modulated by PPAR β/δ and PPAR α, respectively. PPAR β/δ involves in lipid oxidation and adipocyte differentiation [39] while PPAR α regulates lipid metabolism and homeostasis [37]. Our result from this prediction analysis suggests that the reduced level of TG in the TPEE treated group could be associated with the reduced abundance of sequence reads related to SCD-1 in the lipogensis pathway.

At the phylum level, the correlation between Bacteriodetes and the levels of serum HDL, TG, and TCHOL denoted that the abundance of Bacteriodetes in the gut was highly desirable in maintaining healthy blood cholesterol. Contrarily, Proteobacteria phyla have shown unfavorable influence on the serum lipid profile. Clustering analysis based on Ward variance minimization algorithm shows the distinctness of Proteobacteria phylum according to its relationship with the serum lipid biomarkers and liver enzymes. We also found that the correlation between *B. pseudolongum* group and the serum biomarkers provides promising evidence that *B. pseudolongum* group might possess hepaprotective and hypocholesterolemic properties. Previous study has reported *B. pseudolongum* treatment was able to reduce serum TG level and has potential therapeutic implication for the treatment of diet-induced obesity [40].

A healthy gastrointestinal tract can be characterized by the predominant residency of obligate anaerobes like Firmicutes and Bacteroidetes [41]. Major studies have upheld that low F/B ratio denotes healthy gut condition [42] while increased F/B ratio is associated with obesity [43]. A significantly low F/B ratio was observed in Sham group whereas a significantly high F/B ratio was observed in the NC group. Estradiol and TPEE interventions were seen to reduce the F/B ratio in the OVX rats, denoting a reduction in microbial dysbiosis in the OVX rats. Previous study reported that *Lactobacillus rhamnosus* hsryfm 1301, from the phylum Firmicutes, reduced serum lipid levels in the rats [44]. In our serum biomarker analysis, TPEE treatment significantly reduced serum TG levels compared to those in the NC group (Fig. 2).

*A. muciniphila* belongs to the phylum Verrucomicrobia which showed negative correlation with serum TCHOL and LDL/VLDL levels but positive correlation with serum HDL level in Spearman analysis (Fig. 4B). In previous studies, the role of *A. muciniphila* in obesity [45] and diabetes [46] has been reported. *A. muciniphila* was known as a promising probiotic which promote the intestinal health and showing negative correlation with the occurrence of diabetes [43]. On top of that, a collective of studies have reported that *A. muciniphila* plays a crucial role in keeping the intestinal barrier intact, maintaining the permeability of intestinal mucosa and preventing the occurrence of inflammatory response in the host [43]. Therefore, TPEE could serve as a therapeutic option for boosting the growth of *A. muciniphila* to manage inflammatory diseases.

Probiotics are known for their function in the transformation of phytoestrogens, which include daidzein, genistein, formononetin, and biochanin A. The metabolites of these compounds commonly exhibit higher bioavailability, as well as higher estrogenic, antiestrogenic, and antioxidant bioactivities, than their precursors [47]. Formononetin can be converted to daidzein, and sequentially metabolized into equol by intestinal floral; from a pharmacodynamic perspective, this reaction pathway is noteworthy because equol has significantly higher estrogenic potency than its precursor [48]. Kawada et al. Y Kawada, S Yokoyama, E Yanase, T Niwa and T Suzuki [49] previously identified *Lactococcus* sp. strain 20–92, *Slackia* sp. strain NATTS, and *Slackia isoflavoniconvertens* as the bacterial strains found in the human intestine that convert daidzein into equol. Previous study investigated the effects of fermented red clover extract in the presence of a heterogeneous culture of probiotic lactic acid bacteria. Their results showed that 55% of the patients became equol producers after treated with fermented red clover extract for 6 months [18]. These studies suggested that the probiotics in their sample induced the intestinal bacterial milieu of the patients, thereby producing a bacterial composition favorable to equol production. Therefore, TPEE treatment in our study may have introduced a certain amount of gastrointestinal phytoestrogen that in turn potentially increased the abundance of *Lactococcus* sp. in the gut microbiota and potentiated equol production. Besides, *Lactococcus* sp. could be a target for further study because of its ability to convert biochanin A and formononetin into equol and thereby enhance estrogenic activity.

Identifying the composition of TPEE using GC-MS analysis led us to the discovery of 18 functional phytocomponents that could contribute to its bioactivities. To our best knowledge, no previous study has reported the presence of mome inositol in *T. pratense*. Mome inositol possesses cholesterolytic and lipotropic activities [50]. The hypocholesterolemic effect of TPEE reported in this study might be attributed to the presence of mome inositol. It was identified as the major constituent in the methanolic extract of *Corbichonia decumbens* (Forssk.) Exell (*Molluginaceae*) root and stem which was reported to possess anti-inflammatory effects which made it beneficial as an antiulcer drug [51]. Interestingly, the less abundant compounds, ethane, 1,1-diethoxy- and hexadecanoic acid, found in TPEE were reported in previous studies to possess properties which could improve complications related to cardiovascular diseases.

Hexadecanoic acid, another phytocomponent found in TPEE, was previously reported to exhibit hypocholesterolemic effects [52, 53]. From the serum biomarker analysis in this study, TPEE exhibited regulatory effects on the serum lipid biomarkers levels compared to NC group. Ethane, 1,1-diethoxy-, identified in TPEE by GC-MS analysis in this study, have shown to have therapeutic properties such as anti-inflammatory, antipyretic, and antithrombotic effect [54]. The presence of these compounds harmonized with our findings on TPEE’s effect on the serum biomarkers that correspond to cardiovascular diseases. The hypocholesterolemic effects of TPEE may be also attributed to the presence of phytoestrogen which could alter the hepatic metabolism with increased LDL and VLDL removal by hepatocytes [55, 56]. We found that the levels of liver enzymes and biomarkers (AST, ALT, ALP, ALB, and TBIL) were not affected by OVX-induced menopausal condition in rats. The discovery of the major phytocomponent, mome inositol, in TPEE also warrants future research on the mechanism of action of this compound which linked to the regulation of metabolism pathways and gut microbiota composition.

The current study has revealed the potential associations between TPEE treatment and the changes in the gut microbiota composition as well as the changes of lipid profiles in the OVX rats. For future studies, the causation analysis would require fecal bacteria transplantation or potential single strain probiotic identified in this study (*B. pseudolongum* group, *A. muciniphila* and *Lactococcus* sp.) into germ-free host or OVX model. Further investigation on the lipid profile changes and the mechanism of actions in the germ-free or the experimental menopausal host is warranted in the future.

## Conclusions

The current study reported for the first time that mome inositol was identified as the major phytocomponent in TPEE by GC-MS analysis. TPEE intervention potentially ameliorated the gut health and regulate serum lipid levels by yielding lower abundance of sequence reads associated with IBD and PPAR pathways. TPEE treatment also improved intestinal microbiota composition of OVX rats with predominant residency of Firmicutes and Bacteroidetes. Collectively, our analyses indicate that TPEE possesses hypocholesterolemic effects and shows potential for reducing risk factors for coronary heart diseases. TPEE showed complementary effects with PomE on the regulation of serum ALP, ALT, AST, TG, HDL, and LDL/VLDL levels. Therefore, our study suggests that co-administration of TPEE and PomE could be considered as a potential substitute for E treatment to achieve the similar therapeutic effects on the recovery of serum biomarkers.

## Supplementary Information

**Additional file 1. **Species identification of *T. pratense*. The DNA sequencing data obtained was 100% identical to previously reported sequences of *T. pratense* in the genetic sequence database. The dried *Trifolium pratense* was shown on the right.

**Additional file 2. **A diagram illustrating the preparation of *T. pratense* ethanolic extract (TPEE) and the yield of functional indicator components (formononetin and biochanin A).

**Additional file 3. **Timeline of the experiments. A total of seven groups of rats were used in this study, each group consists of 10 animals (*n* = 10). After 1 week of acclimatization, 6 groups of rats were subjected to ovartiectomization and one group was subjected to Sham operation. The operated rats were given one more week for recovery before the commencement of oral administration.

**Additional file 4.** The number of reads and the read lengths for each sample.

**Additional file 5.** Representative images of H&E-stained rat liver were shown. All images were acquired under × 400 magnification. Scale bar: 100 μm.

**Additional file 6.** Alpha diversity indices including rarefaction curves for all the treatment groups.

## Data Availability

All raw sequence reads obtained in this study are available in the NCBI SRA database under project accesion number PRJNA763709. The plant sample datasets generated and/or analysed during the current study are available in the Sequence Read Archive (SRA) repository, accession number: SRR14492033, under the project number PRJNA727441.

## Abbreviations

ALB: Serum albumin
ALP: Serum alkaline phosphatase
ALT: Serum alanine aminotransferase
APOA1: Apolipoprotein A-I
AST: Serum aspartate aminotransferase
c-maf: Transcription factor Maf
E: Estradiol
ELISA: Enzyme-linked immunosorbent assay
GC-MS: Gas chromatography mass spectrometry
GyK: Glycerol kinase
HDL: High-density lipoprotein
HPLC: High performance liquid chromatography
IBD: Inflammatory bowel disease
ILK: Integrin-linked kinase
KO: KEGG orthology
LC-MS/MS: Liquid chromatography with tandem mass spectrometry
LDL/VLDL: Low-density lipoprotein/very low-density lipoprotein
NC: Negative control
OTUs: Operational taxonomic unit
OVX: Ovariectomized
PCoA: Principal coordinate analysis
PC1: Principal coordinate 1
PomE: Pomegranate extract
PPAR: Peroxisome proliferator-activated receptor
RELA: Transcription factor p65
RXR: Retinoid-X receptor beta
SCD-1: Stearoyl-coA desaturase
TBIL: Serum total bilirubin
TCHOL: Serum total cholesterol
TG: Serum triglyceride
TLR4: Toll-like receptor 4
TPEE: *Trifolium pratense* ethanolic extract
