# Supplementary figures and images for: Trifolium pratense ethanolic extract alters the gut microbiota composition and regulates serum lipid profile in the ovariectomized rats
Source: BMC Complement Med Ther. 2022 Jan 4;22:5. doi: 10.1186/s12906-021-03486-w (PMC8725312; doi:10.1186/s12906-021-03486-w)

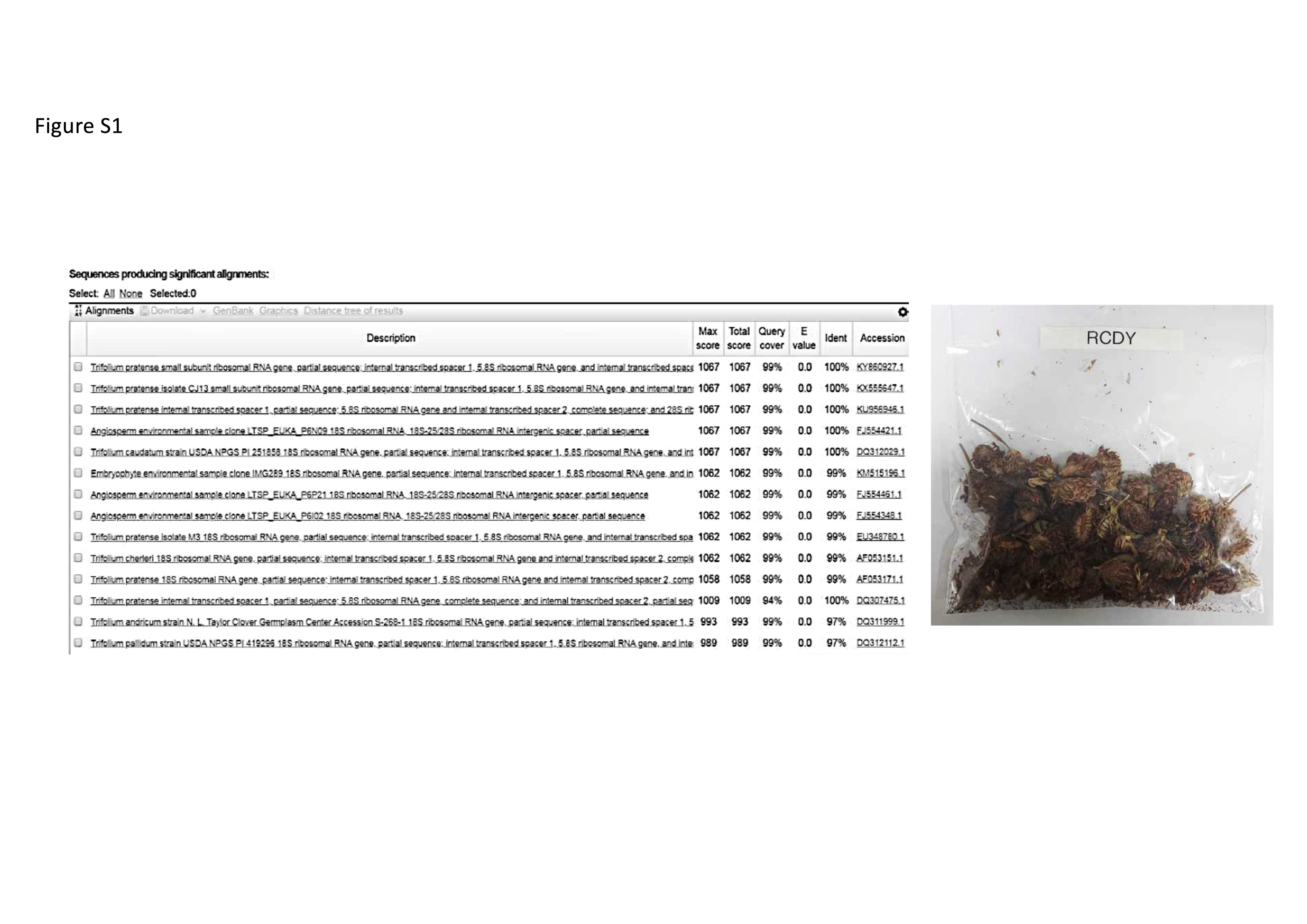

Supplement: Supplementary file 1 — Additional file 1. Species identification of T. pratense. The DNA sequencing data obtained was 100% identical to previously reported sequences of T. pratense in the genetic sequence database. The dried Trifolium pratense was shown on the right. [file 12906_2021_3486_MOESM1_ESM.tiff]

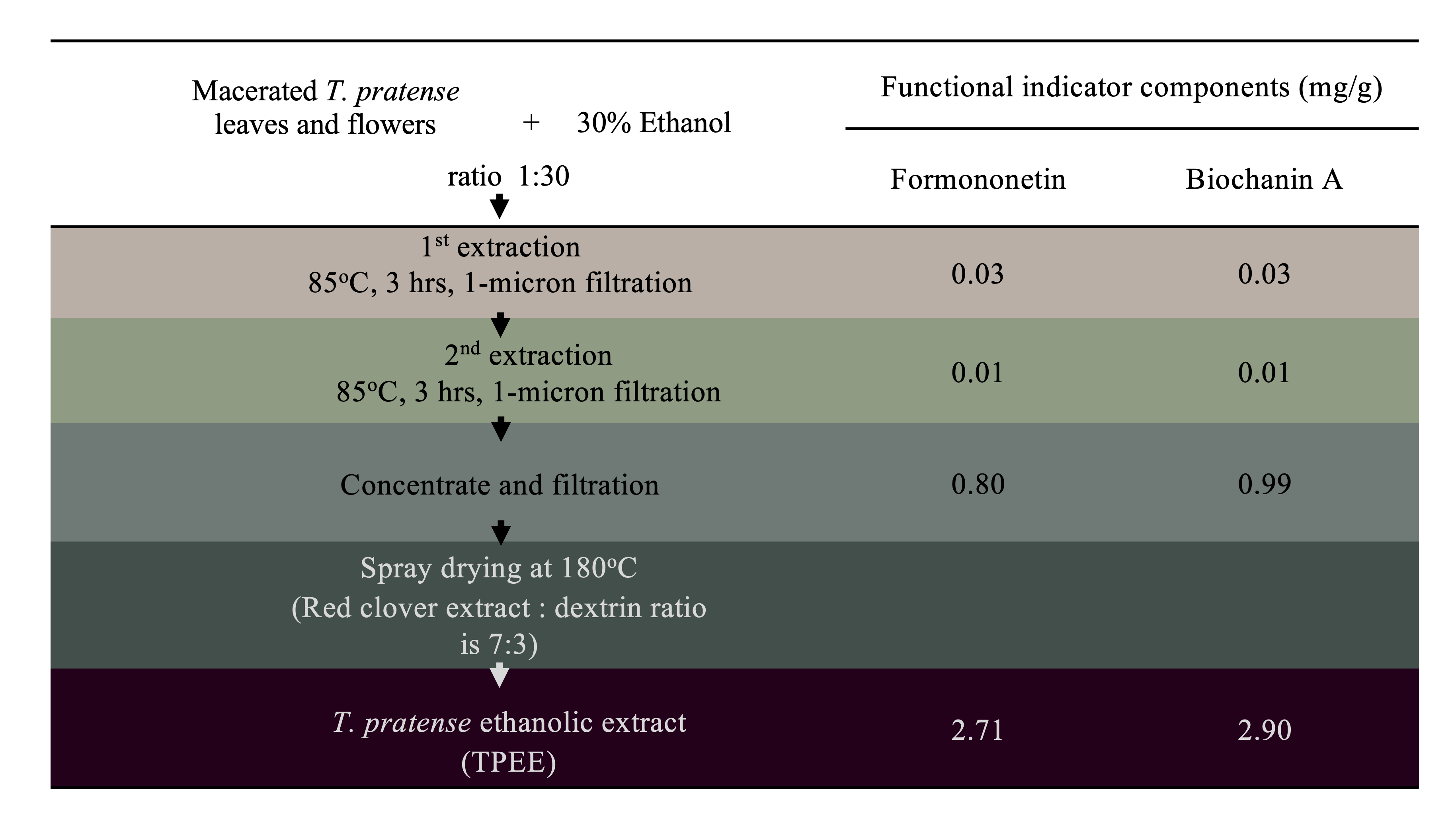

Supplement: Supplementary file 2 — Additional file 2. A diagram illustrating the preparation of T. pratense ethanolic extract (TPEE) and the yield of functional indicator components (formononetin and biochanin A). [file 12906_2021_3486_MOESM2_ESM.tif]

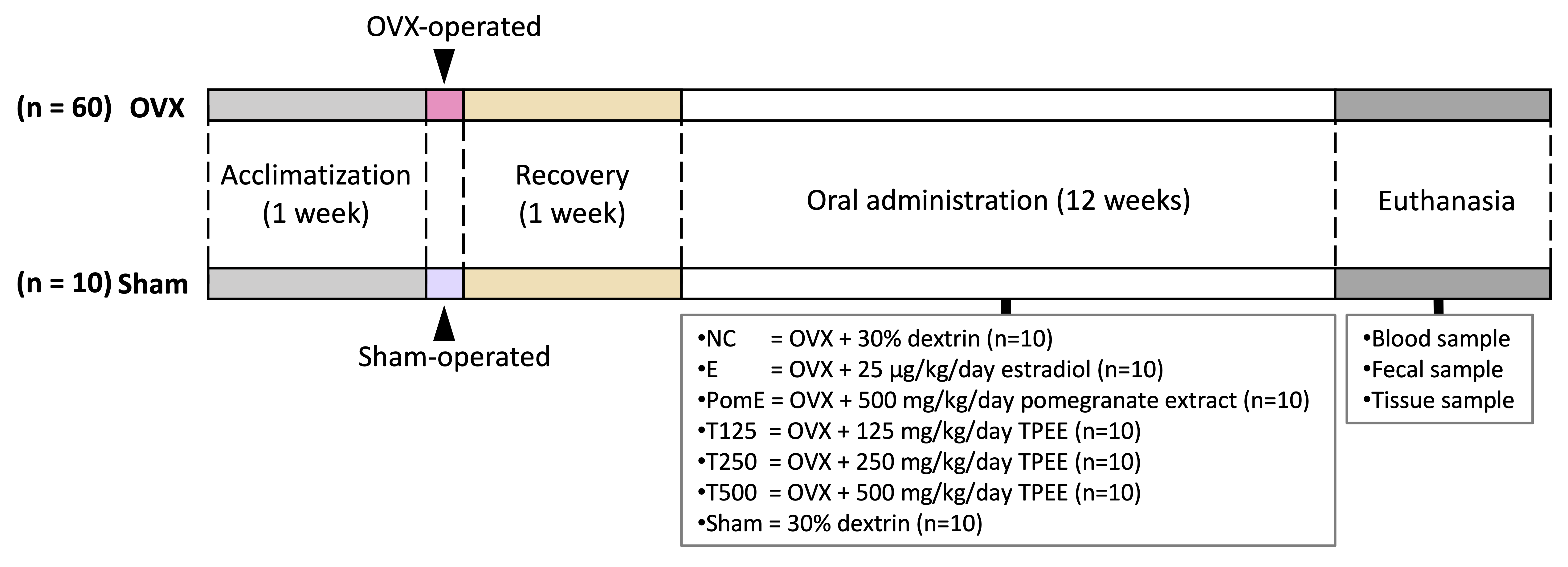

Supplement: Supplementary file 3 — Additional file 3. Timeline of the experiments. A total of seven groups of rats were used in this study, each group consists of 10 animals (n = 10). After 1 week of acclimatization, 6 groups of rats were subjected to ovartiectomization and one group was subjected to Sham operation. The operated rats were given one more week for recovery before the commencement of oral administration. [file 12906_2021_3486_MOESM3_ESM.tif]

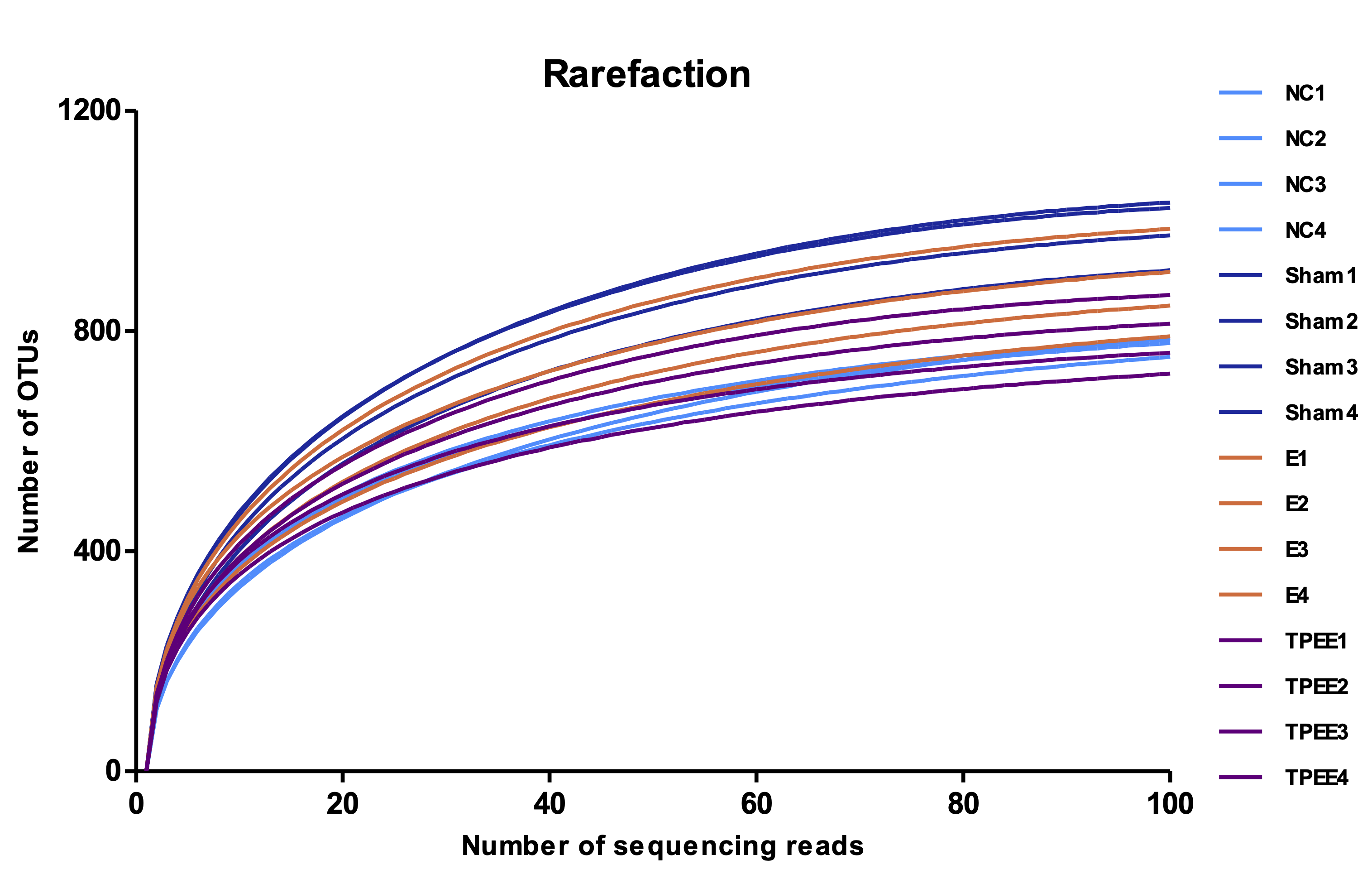

Supplement: Supplementary file 6 — Additional file 6. Alpha diversity indices including rarefaction curves for all the treatment groups. [file 12906_2021_3486_MOESM6_ESM.tif]
