# Supplementary material for: Trifolium pratense ethanolic extract alters the gut microbiota composition and regulates serum lipid profile in the ovariectomized rats
Source: BMC Complement Med Ther. 2022 Jan 4;22:5. doi: 10.1186/s12906-021-03486-w (PMC8725312; doi:10.1186/s12906-021-03486-w)
Supplement: Supplementary file 4 — Additional file 4. The number of reads and the read lengths for each sample. [file 12906_2021_3486_MOESM4_ESM.docx]

Additional file 4. The number of reads and the read lengths for each sample.

| Samples | Total reads | Total valid reads | Removed | | | | Read lengths |
| --- | --- | --- | --- | --- | --- | --- | --- |
|  |  |  | Low-quality amplicons | Non-target amplicons | Chimeric amplicons | Total |  |
| NC1 | 53214 | 50355 | 1331 | 17 | 1511 | 2859 | 446.0 |
| NC2 | 55750 | 52007 | 2167 | 4 | 1572 | 3743 | 444.7 |
| NC3 | 56482 | 52514 | 919 | 1 | 3048 | 3968 | 444.5 |
| NC4 | 60147 | 54500 | 1230 | 2 | 4415 | 5647 | 443.7 |
| Sham1 | 61701 | 51790 | 2285 | 0 | 7626 | 9911 | 446.5 |
| Sham2 | 69716 | 62386 | 1954 | 0 | 5376 | 7330 | 445.6 |
| Sham3 | 81015 | 70222 | 2351 | 0 | 8442 | 10793 | 447.3 |
| Sham4 | 81622 | 69524 | 2293 | 0 | 9805 | 12098 | 449.2 |
| E1 | 50521 | 47883 | 1336 | 10 | 1292 | 2638 | 447.1 |
| E2 | 51391 | 48918 | 1287 | 8 | 1178 | 2473 | 447.0 |
| E3 | 52107 | 47121 | 1239 | 5 | 3742 | 4986 | 444.4 |
| E4 | 60096 | 54532 | 1483 | 4 | 4077 | 5564 | 448.6 |
| TPEE1 | 64284 | 61626 | 1287 | 1 | 1370 | 2658 | 443.1 |
| TPEE2 | 54940 | 53006 | 867 | 3 | 1064 | 1934 | 444.6 |
| TPEE3 | 53052 | 46645 | 896 | 0 | 5511 | 6407 | 444.8 |
| TPEE4 | 52670 | 50605 | 1311 | 4 | 750 | 2065 | 444.6 |
